# Multidisciplinary perspective on a pharmacist-led vaccination clinic in a regional cancer care setting: A qualitative study

**DOI:** 10.1016/j.rcsop.2025.100633

**Published:** 2025-07-05

**Authors:** Kristoffer Johnstone, Joyce Cooper, John Smithson, Beverley Glass

**Affiliations:** a College of Medicine and Dentistry, James Cook University, Townsville, Queensland, Australia; b Cairns Hinterland Hospital and Health Services, Cairns, Queensland, Australia; c College of Medicine and Dentistry, James Cook University, Nguma-bada Campus Cairns, QLD 4878, Australia; d School of Biomedical Sciences & Pharmacy, University of Newcastle, Callaghan, NSW 2308, Australia; e Faculty of Pharmacy, Rhodes University, Makhanda, South Africa.; f School of Pharmacy, The University of Jordan, Amman, Jordan

**Keywords:** Influenza, Pneumococcal, Hospital pharmacy, Immunisation, Cancer, Health professionals

## Abstract

**Background:**

Immunosuppression in patients with cancer increases susceptibility to vaccine-preventable diseases, with suboptimal vaccination rates attributed to complex treatment schedules, timing of vaccination and uncertainty in relation to vaccination effectiveness. While pharmacists are routinely vaccinating patients in the community, high-risk cohorts, such as oncology patients, often lack access to dedicated vaccine services in hospital outpatient settings. Pharmacist-led vaccination clinics offer a promising solution to overcome existing barriers by integrating accessible, trusted healthcare professionals into patients' treatment location, to improve uptake through timely, co-located, and coordinated care. This study aimed to explore the perspectives of healthcare professionals regarding the implementation of a pharmacist-led vaccine clinic in an outpatient oncology unit.

**Methods:**

Semi-structured interviews were conducted with pharmacists, nurses, and doctors from a single site regional referral hospital. Interviews were audio-recorded, transcribed verbatim, deductively and inductively coded and thematically analysed, with emerging themes mapped to the constructs of the Diffusion of Innovation Theory: relative advantage, complexity, compatibility, observability and trialability.

**Results:**

Nineteen interviews were conducted with healthcare professionals, including seven pharmacists, six nurses and six doctors. Factors identified for successful implementation of a pharmacist-led vaccination clinic included patient-centred models, improved convenience and reduced complexity for patients, and compatibility with pharmacists' existing knowledge and role in outpatient units. Barriers were found to be work overload for pharmacists, reduced interaction with general practitioners, and lack of understanding of pharmacists' vaccination training.

**Conclusion:**

Healthcare professionals expressed strong support for a pharmacist-led vaccine clinic in an outpatient oncology unit, recognising the potential to improve vaccination rates. Future research should however focus on assessing patient acceptance of such a service and the impact of such a clinic on vaccination rates.

## Highlights

• Pharmacy vaccine programs are well established in communities, but not in hospitals.
• High confidence in oncology pharmacists' knowledge on vaccines and treatments.
• Pharmacists are skilled to deliver vaccine services but require adequate resourcing.
• Pharmacist-led clinic could be efficient for vaccinating patients with cancer.

## 1 Introduction

Patients with cancer are particularly susceptible to vaccine-preventable diseases (VPDs) due to a compromised immune system, caused by both the disease and the treatment regimens.^1^^,^^2^ Influenza and pneumococcal infections, which are common and can be prevented by vaccination, can lead to severe complications, hospitalisations, and even death, significantly impacting patient outcomes and healthcare costs.^3^

Despite the clear benefits and recommendations to receive vaccinations,^4^, 5., 6. rates among patients with cancer remain suboptimal, with less than 50 % of patients receiving chemotherapy routinely receiving the influenza vaccination.^7^ Several factors have contributed to this, including multiple competing appointments for reviews, scans and treatments, concerns about vaccine safety and efficacy, and limited access to convenient vaccination services.^8^ Additionally, the optimal timing and effectiveness of vaccinations in patients undergoing active cancer treatment remain unclear.^9^

Pharmacist-led vaccination services have emerged to improve vaccination rates in diverse patient populations such as children over 2 years of age, travellers, and for all people eligible under the Australian National Immunisation program.^10^, 11., 12 These services have effectively leveraged pharmacists' expertise in medication management, patient education, and immunisation to deliver accessible and efficient vaccination services. A systematic review demonstrated the effectiveness of pharmacist-led clinics in increasing vaccination rates in community settings,^13^ and patients have overwhelmingly positive experiences with pharmacist-administered vaccinations in community pharmacies, with 95 % willing to return and 97 % recommending the service.^10^^,^^14^ The increased access to vaccinations has improved adherence to recommended immunisation schedules,^15^ and some patients have reported that they would not have been vaccinated without this service.^16^ While community pharmacists are expanding their role beyond that of traditional dispensing, including into general practice, aged care, prescribing and indigenous health,^17^ the scope expansion within hospital pharmacy practice has not kept pace.^18^ Despite their valued expertise and contributions to patient safety, hospital pharmacists often face systemic barriers that limit their scope.^19^, ^20^, ^21^ Many countries, including the United States and Australia, have now authorised all pharmacists to administer vaccines for several diseases, enhancing access for those people who may struggle to access traditional healthcare providers.^12^

Clinical oncology pharmacy practice has developed rapidly over the past several decades with a shift from purely a dispensing model to patient-centred care models. A survey of 4000 members of the European Society of Oncology Pharmacy (ESOP) in 2023 aimed to assess the advancement of clinically focused pharmacy activities into daily oncology practice. Surveyed pharmacists were heavily involved in medication reconciliation, checking for drug interactions, and focusing on pharmacokinetic and pharmacodynamic interactions, with 88 % of their recommendations adopted by oncologists. Oncology pharmacists were also eager to learn and develop to contribute more to the overall outcomes of patients with cancer.^22^ A study considering clinical oncology pharmacy services from the perspective of other healthcare providers found positive perceptions of the pharmacist's role in cancer care.^23^The majority thought they were an integral part of the team to deliver quality patient care as patient educators and delivering services to reduce medication errors.^23^

There is a current knowledge gap about the unique challenges faced by patients with cancer necessitating tailored approaches to vaccination delivery. To offer appropriate vaccination, services need to address the challenges of immune suppression and timing of vaccination,^24^ patient and healthcare professional awareness of vaccination recommendations, and concerns related to potential adverse effects and effectiveness.^25^^,^^26^ A pilot study in older patients with cancer, which included an intervention in the form of an education session with a pharmacist, reported a higher vaccination rate compared to no intervention.^27^ Proposing a new model of care with a pharmacist-led vaccination clinic in an outpatient oncology unit requires engagement and understanding perspectives of different healthcare professionals, like that in other studies which have demonstrated the integration of pharmacists and pharmacy services into primary care.^28^^,^^29^

Studies have identified several factors influencing implementation of novel pharmacy services, including physician relationships, pharmacy infrastructure, patient expectations, staffing levels and external support.^30^, ^31^, ^32^ These elements have collectively shaped pharmacists' ability to implement change.^30^, ^31^, ^32^ Pharmacists are well-recognized for their role in community vaccination programs, however their involvement in vaccinating high-risk populations, including those undergoing cancer treatment, remains understudied.^33^ Therefore, this study aimed to explore the perspectives of healthcare professionals including pharmacists, nurses, and doctors regarding implementation of a pharmacist-led vaccination clinic in an outpatient oncology unit.

## 2 Methods

### 2.1 Study design

A qualitative methodology was employed using semi structured interviews as the data collection method to explore perspectives of health care professionals for a pharmacist-led vaccination clinic in an outpatient oncology setting. To ensure a comprehensive reporting of the study findings, the Consolidated Criteria for Reporting Qualitative Research (COREQ) guidelines were followed to add rigor to this study.^34^

Ethics approval has been granted from Far North Queensland Ethics committee HREC / 2023/ QCH69788 (Apr Ver. 3) – 1628.

### 2.2 Study setting and sampling

This single site study involved healthcare professionals including pharmacists, nurses and doctors that have worked or currently working in the Cairns hospital (a large 771 bed public regional referral hospital) in the ambulatory outpatient oncology unit.

Interview recruitment utilised a combination of purposive and snowballing sampling to reach a diverse pool of participants. Department wide emails and research presentations at staff meetings and journal clubs were undertaken to encourage participation. Emails were sent to pharmacists, nurses and doctors who have worked in Cairns' outpatient unit for a minimum of 3 months with direct involvement in the care of patients with cancer. This email explained the study, inviting interested individuals to contact the research team. Participating staff were encouraged to refer appropriate colleagues to the research team.

### 2.3 Theoretical framework

Roger's Diffusion of Innovation (DOI) theory^35^ is one of the most widely used models to understand adoption of change, with more than 51,000 citations in the literature over the past 50 years.^36^ Guided by this theory, participants' perceptions of the innovation i.e. the pharmacist-led vaccination clinic was explored against the constructs of relative advantage, complexity, compatibility, observability and trialability and the factors influencing the adoption or rejection of this pharmacist-led clinic.^20^ This study examined the role of communication channels, and institutional, personal, and social barriers in the diffusion process. Expanding the pharmacist's role in the hospital outpatient setting to include a vaccination clinic is an expansion on the hospital pharmacist's current scope of practice and thus the DOI theory can be used as a framework to understand this transition. DOI theory has been widely used in many disciplines to explore innovations in healthcare^37^ along with pharmacy practice, including vaccination services.^38^^,^^39^

### 2.4 Data collection

Considering the exploratory nature of this research, a semi structured interview was the selected method.^40^ Questions were informed by Rogers' DOI theory^33^ with follow-up prompts to provide more flexibility and freedom to understand views on vaccinations and the implementation of a pharmacist-led vaccination clinic.^20^^,^^41^^,^^42^ Initially, participants answered questions on their practice and experience. They were then asked to discuss their experience with influenza and pneumococcal vaccination in patients with cancer and answer questions around the potential for a pharmacist-led vaccination clinic in this setting.

To ensure the interview guide was robust, a pilot interview was conducted. This pilot interview allowed for the evaluation of the interview guide's clarity, flow, and ability to prompt participants to share their in-depth experiences. Additionally, the pilot interview served as a valuable learning experience for the researcher, fostering reflexivity by highlighting any potential biases or areas where further interviewer training might be necessary. Based on the pilot interview findings, minor modifications were made to the interview guide to optimise its effectiveness in capturing the desired data.^43^ (Interview guide included as supplementary material, Appendix B).

Interviews were then conducted with pharmacists, nurses, and doctors between February to May 2024. All interviews were conducted by the first author in person or online using Microsoft Teams ™. Interviews were digitally recorded and transcribed verbatim. All identifying information was removed at the time of transcription. Participant recruitment and interviews continued until data saturation with no new themes emerging.

### 2.5 Data analysis

Qualitative thematic analysis was employed, supported by the digital program NVivo ™ to analyse interview transcripts. This involved several phases: familiarisation with the data, generating initial codes, identifying themes, reviewing the themes, defining and naming the themes, and production of report including selection of illustrative data and patient quotes.^44^ The transcripts were condensed and coded by two coders (KJ and BG) independently to address potential bias of the primary coder (KJ) with any discrepancies resolved by discussion with a third member of the team (JC). These codes were then categorised based on existing constructs from Rogers' DOI theory.^45^ Inductive coding was used to identify barriers. A reflective understanding of prior knowledge was applied, and constant comparative analysis was utilised to refine themes, identifying relationships among them, and construct overarching categories and themes.^46^^,^^47^

### 2.6 Reflexivity

The research team comprised of KJ (a Research Master's student), JC, JS and BG who are experienced researchers in qualitative pharmacy practice. Reflexivity is a critical aspect of qualitative research, involving a researcher's conscious awareness and examination of their own biases, values, and assumptions. By acknowledging and reflecting on these personal factors, researchers can strive to minimise their influence on the research process and interpretations. Through reflexive practices, peer review, and critical self-reflection, the researchers strove to enhance the credibility and trustworthiness of their findings. Given the team's diverse experience including in hospital pharmacy (JC), cancer unit staff member (KJ), and community pharmacy vaccination pilots (BG), a multi-step analysis was employed to mitigate potential bias. To validate the interview guide, a pilot interview was conducted. By reviewing the pilot interview, the researcher was able to identify potential bias and refine the interview questions.^40^

## 3 Results

Nineteen healthcare professional interviews were conducted with seven pharmacists, six nurses and six doctors (participant characteristics Table 1). Four interviews were conducted via Microsoft Teams ™, and fifteen face-to-face with the interview length ranging between 9 min to 26 min with an average duration of 12 min.

**Table 1:** Participants' characteristics.

	Pharmacist	Nurse	Doctor
	*N* = 7	*N* = 6	N = 6
Gender			
Male	1	-	3
Female	6	6	3
Years experience			
<10	3	1	-
10–20	4	1	4
>20	-	4	2
Years experience in cancer care			
<10	6	1	3
10–20	1	5	1
>20	-	-	1
Cancer centres worked			
Metro	2	2	6
Regional	7	6	6
Rural	-	2	2
Remote	-	-	1
Experience with vaccination			
Administration	-	4	1
Education	6	1	6
Prescribing	-	-	2
Recommendation	6	-	6
None	1	1	
Think patients are up to date with vaccinations			
Yes	3	2	1
No	3	2	4
Unsure	1	2	1
Support for a pharmacist- led clinic			
Positive	7	5	5
Negative	-	1	1

* Participants could answer yes to more than one option so the numbers in each section might exceed the total number of participants.

Most participants acknowledged the need for appropriate vaccination prior or during cancer treatment, but did not know whether the patients were up to date with vaccines as it was not a “priority” and they were time-poor. Majority of participants thought that someone outside the oncology unit should be responsible for providing vaccination services, for example the General Practitioner (GP) or that the patient themselves needed to be proactive in identifying their immunisation requirements.

The DOI theory, a well-established framework for understanding the adoption of new ideas or technologies, was employed to analyse healthcare professionals' perspectives of a pharmacist-led vaccination clinic for patients with cancer, by examining the five core attributes of Rogers' DOI- relative advantage, compatibility, complexity, trialability and observability.^35^ While participants expressed overall approval of the concept, inductive coding resulted in the emergence of a theme, which depicted the barriers, which might impact the implementation of an appropriate model for vaccination of patients with cancer.

The following sections describe themes emerging on the potential implementation of the pharmacist led-vaccination clinic mapped to the constructs of the DOI Theory,^35^ in addition to the barriers to the implementation of this innovation which emerged because of inductive coding of the data, with quotes assigned to doctors (D), pharmacists (P) and nurses (N), respectively.

### 3.1 Relative advantage

Rogers defines “relative advantage” as the degree to which an innovation is seen as superior to existing alternatives.^35^

All healthcare professionals expressed a positive sentiment towards the introduction of a pharmacist-led vaccination clinic in an outpatient cancer unit. They perceived this approach as advantageous compared to existing options for patients with cancer seeking timely and appropriate vaccinations. The vaccination clinic's perceived benefits, particularly increased convenience with fewer visits to clinics for patients with cancer were seen as the primary drivers for its potential successful adoption.^20^

“*I think patients on regular treatment would like to get it all in house because they are already here. For patients on follow up a lot of time they might see their GP who recommends a vaccine but might wait 3–6 months to see the specialist to ask if its ok, then have to go back to the GP and then get it*. “(D6).

The skills and knowledge of pharmacists working in cancer units was acknowledged, with their up-to-date knowledge of treatments and vaccines giving confidence to doctors and nurses of providing a safe service, with appropriate vaccination.

“ *I think I would feel a lot more comfortable because a lot of times GPs themselves aren't sure about what vaccines can and can't be given, so often they just won't”* (D4).

“*I think it would be amazing and appropriate. I think we answer a lot of questions as the information is not easy to find. So, I think having it all facilitated by someone who know all about it would be very useful.*” (P3)

### 3.2 Compatibility

“Compatibility” refers to the degree to which an innovation aligns with the values, experiences, and needs of potential adopters.^35^

The proposed pharmacist-led vaccination clinic aligns with the practice of community pharmacists giving vaccines as part of their current role. Many said it “*makes sense*” as its already widely accepted and “*people can't remember when it wasn't the case*”. The consensus was that it was a logical use of pharmacist's skills and should be widely accepted as standard of care.

“ *it would be comparable that happens in community with patients receiving vaccinations from community pharmacies which is well established*” (D3).

“ *I personally have just had my flu and had all my COVID vaccines in the community pharmacy. I had good rapport, I haven't had any issues, they done all the right checks, so I have had a good experience*.” (N3).

Most doctors supported that the model for vaccination used in community pharmacy could also be applied in hospital pharmacy and saw little concern with the safety of pharmacists recommending vaccines to their patients without direct oversight.

“*if there was a pharmacy led clinic whereby I don't need to see is it worth it, or the patient themselves refer. And then the pharmacists communicate to the GP plus specialists”* (D1).

Pharmacists thought this role could easily be included into their current tasks which include taking medication histories and counselling patients prior to and during treatment.

*“ I think it could work around easily as well. Having pharmacists as vaccinator would increase the likelihood of the pharmacist being particular mindful of vaccination status as part of their chemotherapy screening and will identify more patients to offer vaccination” …………. “ I guess it should be tacked on to an existing thing [appointment] rather than a completely new thing that results in more work for others”-* (P5)

### 3.3 Complexity

“Complexity” refers to the perceived difficulty of understanding and using an innovation, with innovations that are simple and straightforward more likely to be adopted.^35^

There was general acknowledgement that a pharmacist-led vaccination clinic was a simple extension of existing roles performed by pharmacists in the oncology unit and like pharmacists administering vaccines in the community.

“ *the pharmacy opposite the hospital do the same things, it's quite common and I don't see the difference, I daresay I don't see much of a challenge with it.”* (D2).

There were thought to be some additional benefits to pharmacists taking the leadership for vaccines as the hospital pharmacy service already manages post bone marrow transplant vaccines and that this would simplify some processes.

*“Pharmacy a lot of times are the ones dealing with the prescriptions and you need to know as part of a protocol as to when its safe and when its note. So, I think if there's anyone that knows a lot about it and when it would be safe to give would be a pharmacist”* (N1).

There was further benefit seen with reduced complexity and improved transparency with better documentation and recording of vaccinations, if this was managed by the pharmacy service.

“ *the AIR (Australian Immunisation Register) documentation is very poorly done as a general comment. That's the other benefit we would have some control for want of a better word about what's being done and recording AIR*” (D6)

### 3.4 Trialability

Rogers defines “trialability” as the extent to which an innovation can be tested and modified. The ability to pilot a medical intervention on a limited scale allows clinicians to view its feasibility, patient acceptance and potential outcomes.^35^

Participants favoured a trial of the service to identify how the service would flow, the additional demand it might place onto the pharmacy team and how issues that might arise may be resolved. There were differing opinions on the best patient group to use as the trial patient cohort, which varied from type of diagnosis, vaccines for patients post-transplant, days of the week, or focus on a single vaccine.

“*I don't know how much additional work it would create, because if we are taking on the patients that we mostly do vaccinations [for currently which] are post-transplant haematology patients. The patient cohort is not huge numbers*.” (P4).

“ *You'd pick a patient group or like a risk group, or you could pick a tumour stream then use that as your cohort. I guess that to see if it's feasible on a small scale*.” (D5)

### 3.5 Observability

“Observability” is the degree to which the results of the innovation are visible to others.^35^

The most observable benefit cited was related to patient perspectives and their utilisation of a service.

“*Definitely patient feedback with the patient experience, you could ask them how were they previously managing their vaccination requirements”* (P4).

Doctors and nurses would like to see impact on infection rates and admission rates with the infectious complications of patients with vaccination versus not vaccinated but acknowledged that this would be a very large undertaking.

“*I don't know if there is something you would look at to say do they present with more hospital admissions and complications. But that's a big study right?”* (N6).

“ *it would be good to know, it's pretty hard to capture the percentage of patients with uptake in vaccination and dig up how many patients had flu or COVID. Not easy to do and then uptake and utilisation of the service*” (D1).

### 3.6 Barriers

Although the majority of participants were positive about the implementation of a new pharmacist-led clinic, there were some barriers to implementation raised: concerns about additional workload on an already overworked pharmacy team, and that this clinic might take away from other cancer treatment related tasks performed by pharmacists.

*“ if staffing and time allowing it, I can't think of anyone who would have concerns about this unless it was impacting on our ability to do core business.*” (D3).

Concerns were raised about potentially compromising the patient- GP relationship, as preventative health management, such as vaccinations, is often considered a core responsibility of general practitioners and not of the oncology centre.

“ *GPs are the ones who know their patients and where they are up to, what other risk factors outside of cancer setting” …………..“vaccinations are important in our population, but we shouldn't become their primary care [provider] and that has to be quite clear. That is not what the goal of the oncology centre is*” (D5).

While most participants understood that community pharmacists could work independently and prescribe and administer an increased range of vaccines, some doctors would still like to be consulted about vaccines for their patients.

*“ because now you are talking about initiation, the prescription and administration, presumably autonomously, separate to medical. I would favour a shared decision making around that”* (D6).

There were concerns that the pharmacist-led model might introduce unnecessary complexity if the pharmacist does not have full control over the entire vaccination process.

“ *but if we are not doing the administration, then they're needing to coordinate with other colleagues and that will potentially pose some barriers and obstacles”* (P3).

All participants acknowledged that appropriate space and resources would be a barrier for trialling or implementing a new vaccination service. Existing public pharmacy workspaces, that included hallways, wouldn't meet the pharmacy or hospital consulting room legislative requirements to conduct private consultations for vaccination.^48^^,^^49^ The existing fridges for housing chemotherapy also does not meet the stringent vaccine fridge requirements,^50^ which could impact stability and effectiveness of vaccines. Resourcing was routinely mentioned, with all HCPs acknowledging pharmacy was not adequately staffed or resourced to currently facilitate the service.

*“I guess the challenges that we would face would be around again staffing, just having the people with the time to do this and potentially the space as well.”* (D3).

While most participants thought pharmacists had the knowledge of the appropriate vaccines and the timing regarding treatment, pathology results and response, there were some concerns about the skills required to administer and manage complications of vaccine administration.

“*upskilling of certain people, whether pharmacists do actually administer vaccines, we will need to upskill*” (P3).

## 4 Discussion

This study investigated the perspectives of healthcare professionals regarding the establishment of a pharmacist-led vaccination clinic in an outpatient oncology setting. To develop a successful and sustainable new model of care with full scope for pharmacists to be utilised in vaccination services, its crucial to understand the perceptions of key stakeholders. Perceived enablers and barriers to the implementation of a pharmacist-led vaccination clinic have been identified. HCPs were positive acknowledging the potential benefits of this model for improving vaccination rates among patients with cancer. All HCPs in this study focused on the relative advantage of a pharmacist-led model to provide a vaccine service for patients undergoing cancer treatment, recognising the expertise of oncology pharmacists in managing the complexities of cancer treatment and vaccination, particularly regarding the safe and effective administration of vaccines alongside anticancer therapies. Additionally, HCPs emphasised the role of pharmacists in enhancing vaccine information transfer through improved data recording within a national health database, the AIR.

The DOI theory suggests that stakeholders must perceive a relative advantage in adopting an innovation^20^ like a pharmacist-led vaccination clinic in an oncology setting. Successful implementation requires stakeholders to recognise this program offers significant benefits such as improved accessibility, enhanced care, or increased vaccination rates compared to existing pathways.^4^^,^^20^ An emerging theme in the study was the perceived relative advantage of a pharmacist-led vaccination service in the oncology setting. Participants suggested that such a model could offer improved accessibility and integration with cancer treatment workflows, potentially addressing current gaps in vaccine delivery. GPs expressed concerns about their suitability of administering vaccines to patients undergoing cancer treatment and acknowledged potential limitations in their current knowledge regarding appropriate vaccination for this specific cohort.^25^^,^^51^ The doctors felt that pharmacists trained in oncology had the clinical expertise to identify when vaccination was suitable for administration in patients on treatment, which supports that healthcare professionals have high confidence in the ability of oncology pharmacists to deliver advanced services.^52^^,^^53^

A pharmacist-led vaccination clinic within a hospital setting demonstrates strong compatibility with existing knowledge of pharmacy-based vaccination services in the community. The proposed model shares similar objectives with the community-based model, which has been expanded to meet the growing demand for vaccination.^10^^,^^54^ Oncology pharmacists have shown significant interest in developing more skills in supportive care for patients with cancer, which would optimise vaccination for patients with cancer.^55^ HCPs have suggested that hospital pharmacist-led services would align with the public's expectations of vaccinations in community pharmacy,^54^ which is well supported in the literature.^56^^,^^57^ In support of increasing pharmacist's involvement in vaccination, studies have shown pharmacists can have statistically significant impact on vaccination uptake, particularly for influenza and pneumococcal vaccines, consistent with the current study.^58^ This study highlighted that with appropriate staffing, it was seen as an appropriate extension of existing pharmacy services in an oncology day unit. This similarity and compatibility with existing workflows enhance the likelihood of uptake and referrals by current healthcare professionals working in oncology.^59^

Complexity of a new pharmacist-led vaccination model will impact the rate of adoption.^20^ Simplifying the process for patients to receive vaccinations and ensuring the model is grounded in evidence-based practices will enhance its acceptance and integration into healthcare systems.^60^ The study found that the proposed model will reduce the complexity of current pathways to get vaccinated, with patients able to get a complete in-house service without the need for back and forth between multiple different health professionals. Participants also suggested that pharmacists will improve the transparency of vaccine documentation, facilitating smoother transitions of care and ensuring timely information sharing. Clinical documentation improvement (CDI) has been shown to enhance effectiveness, safety, and reduce healthcare costs.^61^ This reduced complexity of streamlining documentation and patient handovers was perceived as an important factor that would likely increase the adoption of this new model of care.^59^

Participants thought a pharmacist-led clinic model could be trialled with either a single vaccine or a single patient diagnosis cohort. During the trial it was suggested that patient feedback was a key factor to monitor, along with the overall utilisation of the service, and the potential to observe patient outcomes such as infection rates. Potential barriers to successful implementation of pharmacist-led vaccination clinics in the outpatient oncology centre included limited understanding of hospital pharmacists' training and the ability to administer vaccines, an understanding that did not align with HCPs perception of community pharmacists,^62^^,^^63^ with multiple agencies strongly advocating for increased funding to improve patient vaccine access.^64^

Concerns were raised that pharmacist-led services might disrupt usual preventative health pathways due to potential gaps in follow-up with a patient's regular GP. A recurring view held by the HCPs in the oncology unit was that vaccination was someone else's responsibility and appears to stem from perceived lack of role delineation and role ambiguity. While all HCPs acknowledged the importance of vaccinating cancer patients, many did not see it as their core responsibility. Cancer specialists believed that vaccination is a GP role and previous literature has highlighted that fragmented care increases patients' reliance on cancer centres to manage all their care while on treatment.^25^ Literature has also suggested that patients have reduced follow up with their GP, while on treatment, and oncologists have increased requests for primary care.^65^ Specialist doctors acknowledged that GPs frequently awaited oncologist/haematologist approval before proceeding with routine vaccination, leading to delayed implementation. This highlights ongoing issues with communication between the HCPs in the hospital and primary care. By taking the lead for vaccination in a pharmacist -led clinic in oncology units, pharmacists can help streamline and improve this continuity of care.

While most specialists thought that pharmacists could vaccinate autonomously, they still would like to be part of the decision-making process. They suggested that there could be more collaboration between cancer specialists, GPs and pharmacists in delivering vaccination to patients with cancer. Research has suggested that patients would like their GP involved in their cancer management and supportive care and this could also be supported with improved communication via the pharmacist.^66^

As a result of the COVID-19 pandemic, there has been a shift to the involvement of pharmacists in all treatment modalities and personalised medicine.^55^ Hospital pharmacists working in the oncology area need to be utilised for their therapeutic knowledge of vaccines and contemporary treatment for cancers which includes targeted therapies and immunotherapy.^67^ Hospital pharmacists have advocated for better public awareness of the their roles, especially being part of the multidisciplinary cancer care team in supporting patients during treatment, which could include vaccination.^55^ Study results found that with appropriate staffing, providing vaccinations was seen as an easy extension of existing pharmacy services in an oncology day unit. Alongside findings from previous studies, the research identified potential barriers, particularly an increase in pharmacist workload, to implementing pharmacy-led vaccination services.^62^^,^^63^ Addressing these barriers requires a coordinated approach to streamline communication, secure sustainable funding, and expand pharmacists' scope of practice. Overcoming these obstacles could significantly enhance vaccination accessibility, particularly for high-risk populations like patients with cancer.

## 5 Strengths and limitations

The strength of this study is the diverse healthcare professionals' perspectives that were purposively recruited to capture all relevant views and reached data saturation. Furthermore, the questions were designed according to an extensively researched framework and was piloted with in-depth probing questions. Despite the strengths, limitations have been identified. Limitations to this study include a single site study, limiting generalisability of the findings. In addition, participants, although purposively sampled might have been biased by their interest in or knowledge of the topic and therefore not representative of all healthcare professionals working with patients with cancer or in a cancer unit. By employing semi-structured interviews informed by a well-established theory, balancing the participants from each discipline, conducting a pilot interview, enhanced rigor via reflexivity through regular team meetings to address potential biases and interpretations of emerging themes from the transcripts, and the use of direct quotations to support findings were employed to mitigate bias.

## 6 Conclusion

This exploratory study presents a pharmacist-led vaccination clinic as a strategy to increase vaccination rates within an oncology setting. Recognising the multidisciplinary nature of oncology care, including pharmacists, doctors, and nurses, this study highlights the importance of their perspectives for the successful implementation. HCPs strongly supported a pharmacist-led vaccination service, acknowledging oncology pharmacists' expertise in both vaccines and cancer treatments. Building on the positive reception of prior community pharmacy vaccine programs, this study identifies key implementation strategies: ensuring appropriate training for hospital pharmacists in vaccines administration and hypersensitivity management, allocating sufficient pharmacy resources, improving pharmacy infrastructure, and enhancing communication with GPs. Future research should evaluate the implementation of a pharmacist-led vaccination clinic in an oncology setting, assessing impact on vaccination rates, patient satisfaction, and perceived value. Additionally, a feasibility study including cost-effectiveness analysis would provide insights into both scalability and sustainability.

## CRediT authorship contribution statement

**Kristoffer Johnstone:** Writing – review & editing, Writing – original draft, Visualization, Software, Methodology, Investigation, Funding acquisition, Formal analysis, Data curation, Conceptualization. **Joyce Cooper:** Writing – review & editing, Supervision, Methodology, Formal analysis. **John Smithson:** Supervision, Methodology, Conceptualization. **Beverley Glass:** Writing – review & editing, Supervision, Methodology, Formal analysis, Conceptualization.

## Funding

The primary author declares they have received funding support from 10.13039/100010230Queensland Health as part of an education grant to support publication costs.

## Declaration of competing interest

The authors declare the following financial interests/personal relationships which may be considered as potential competing interests:

Kristoffer Johnstone reports article publishing charges was provided by Queensland Health. If there are other authors, they declare that they have no known competing financial interests or personal relationships that could have appeared to influence the work reported in this paper.

## Data Availability

The datasets generated during and/or analysed during the study are available from the corresponding author on reasonable request. Some data may contain information that could potentially be linked to participants. Access to these data will be granted upon approval of a data access agreement that ensures the confidentiality and ethical use of the data.

## Appendix A Supplementary data

Supplementary material Image 1
