# Supplementary material for: Multidisciplinary perspective on a pharmacist-led vaccination clinic in a regional cancer care setting: A qualitative study
Source: Explor Res Clin Soc Pharm. 2025 Jul 5;19:100633. doi: 10.1016/j.rcsop.2025.100633 (PMC12275892; doi:10.1016/j.rcsop.2025.100633)
Supplement: Supplementary file 1 — Supplementary material [file mmc1.docx]

**Appendix A** Consolidated Criteria for Reporting Qualitative Research (COREQ) guidelines.

| 1. Interviewer/facilitator | KJ- first author |
| --- | --- |
| 2. Credentials | BPharm, Graduate Diploma in Public Health and Tropical Medicine, FANZCAP, MPhil (Health) candidate |
| 3. Occupation | Pharmacist |
| 4. Gender | male |
| 5. Experience and training | Over 10 years of cancer experience, post graduate studies and undertaking higher research degree |
| 6. Relationship established | Yes - colleagues |
| 7. Participant knowledge of the interviewer | Colleagues- work in the same unit and knew research was associated with master’s degree |
| 8. Interviewer characteristics | Bias – as part of the interview schedule it was discussed that these views are their own and should not be influenced by what they thought the interviewer would want to hear.  As a pharmacist, it could be perceived of wanting to have a positive influence on pharmacists expanded roles. |
| 9. Methodological orientation and  Theory | Interview guide and analysis= Diffusion of Innovation Theory |
| 10. Sampling How were participants selected? | Purposive sampling and snowballing |
| 11. Method of approach | Direct and broad email recruitment to department along with presentations at stream specific meetings. |
| 13. Non-participation | Not accounted for |
| 14. Setting of data collection | Hospital clinic room or via Microsoft teams virtually |
| 15. Presence of non-participants | No one else present during interviews, all interviews were recorded. |
| 16. Description of sample | There was wide range of experience in most professions, ranging from a couple of years to over 30 years. It was a general representative of the gender make up of health professionals with even split of male and female doctors and more female pharmacists and nurses. |
| 17. Interview guide | Interview guide is provided with introduction statement, questions and follow up prompts.  The interview schedule was piloted and reviewed by senior researcher. |
| 18. Repeat interviews | n/a |
| 19. Audio/visual recording | Yes- the interviews were recorded via audio recording when conducted face to face and video and audio when conducted over Microsoft teams ™ |
| 20. Field notes | Minimal field notes were used |
| 21. Duration | The interviews ranged from 9 minutes to 26 minutes with an average of 12 minutes |
| 22. Data saturation | Data saturation is discussed when no new themes emerged |
| 23. Transcripts returned | No- all transcripts were clear in recording and no clarification required. |
| 24. Number of data coders | 1 primary coder with 2 secondary coders to check for consistency |
| 25. Description of the coding tree | no |
| 26. Derivation of themes | Themes were identified in the literature in advance according to DOI and coded to a known framework |
| 27. Software | NVIVO ™ was used for analysis |
| 28. Participant checking | no |
| 29. Quotations presented | Quotes have been provided for all themes |
| 30. Data and findings consistent | There were consistent findings in the data for most themes but some conflicting results which gave a good overall presentation of the data and alternative views. |
| 31. Clarity of major themes | Major themes have been presented in the results for the constructs of DOI  Inductive coding of barriers |
| 32. Clarity of minor themes | Minor themes have not been coded due to the word limit restrictions and grouped into major themes. |

**Appendix B -Interview schedule**

**Introduction, research topic and confirming consent**

“Thank you for taking the time to speak with me today. (My name is kris, if they are unknown to me) and I'm a pharmacist at in the liz plummer cancer centre. I'm conducting this interview as part of my research on influenza and pneumococcal vaccines in cancer patients. Your participation is entirely voluntary, you have the right to withdraw from the interview at anytime without consequences. I am confirming you have read the information sheet, signed the consent form and still happy to proceed with the interview?” (Visualise signed consent)

**Process for the interview**

“I'll be recording our conversation so that I can transcribe it later. I will also be taking some notes through out the interview. I'll keep your name and any other identifying information confidential. I know you're busy, so I'll try to keep this as brief as possible.”

**What will be in the interview**

“I'll start by asking you some general questions about your experience with cancer patients and vaccination. Then, I'll ask you some more specific questions that are related to my research questions about a pharmacist led vaccination clinic. This is to gain insight from multiple different health professional perspectives. I may ask for further information about your statements or just ask for clarification to ensure I have interpreted it correctly. If you have any questions at any time, please don't hesitate to ask.”

**Starting the interview** -“We are going to start with the interview questions shortly, I am going to start recording now”

**Demographic questions**

- “What is your role in the hospital or the unit?”
- “How many years’ experience do you have working, and how many years in cancer care setting?”
- “How many different cancer centres have you worked in?” “Were they regional, remote or metro?
- “Do you have experience in cancer patient vaccination with influenza and pnuemococcal? This could include administering or recommending vaccines?
- “In your experience, are patients up to date with their vaccinations?”

**Question 1: “What are your thoughts on a pharmacist-led vaccine clinic in the cancer care unit?”**

Proposed clinic = pharmacists reviewing patients need for vaccinating and then potentially administering them or prescribing for nurses to administer.

Prompts

-Is there an unmet need currently?

-How does this compare with existing models?

-What do you think about Patient perspectives

**Question 2: “How do you see this service fitting with the existing model of care?”**

Prompts -

- What would not make it compatible? (Skills, space, time)
- What do you think about Staff (nurses, doctors, pharmacists) perspective
- Would there be any conflict between different HCP?

**Question 3: “Is implementing a new service model such as this pharmacist-led vaccination possible at the hospital – Why /Why not?”**

Prompts

- Institutional barriers
- Health professional barriers? Such as the role of the pharmacist or role overlap/conflict? Any conflict between HCP?
- Patient barriers? Would patients accept pharmacist recommendations or pharmacist administering vaccines.

**Question 4: “Do you think the proposed model could be trialled before introduction?”**

Prompts

- Is it worth trialling? Why or why not?
- Any suggestions on how to trial it?

**Question 5: “What could be observed to assess the trial for success or failure?”**

Prompts

- Measurable outcomes?
- Patients factors (rates of vaccination, satisfaction)
- HCP – doctors and nurses vs pharmacist surveys?

**Interview conclusion script**

“That concludes the interview questions today, is there anything else you would like to add or share?

I would like to extended my thanks for taking the time to answer my questions during todays interview. Your responses will provide us with valuable insights into patient vaccination and the perceptions of the pharmacists role.

As we move forward with the research, we will carefully analyse the responses from all participants. Each participants responses play a crucial role in shaping the outcomes and conclusions of this research so your input is extremely valuable. We kindly request that you do not share the content of todays interview with anyone else to ensure that all participants have an equal opportunity to provide their perspectives. Confidentiality is vital to maintaining the integrity of research.

Once the research is complete, we will be sharing a copy of the outcome with everyone who participated and the department.

Thank you again for your willingness to participate in this research. Your expertise and insights are instrumental in helping us understand our patients and staff to improve our services. If you have any questions about the research today, please feel free to reach out to me at any time. “

**Cues to use throughout the interview**

**Clarify**

“Can you give me an example?”

‘What do you mean by that?”

“it seems like you are saying… (repeat their words back to them)”

**Expand**

“Can you explain that in more detail?”

‘What else can you tell me about that?”

**Redirect**

“We can come back to that, I would like to focus on your experience with…. (previous topic)”

“I am following you, but I am not sure how this relates to our topic, can you help me understand the connection?
